# Access to healthcare for people with disabilities in South Africa: Bad at any time, worse during COVID-19?

**DOI:** 10.4102/safp.v63i1.5226

**Published:** 2021-07-19

**Authors:** Emma L. McKinney, Victor McKinney, Leslie Swartz

**Affiliations:** 1 Interdisciplinary Centre for Sports Science and Development, Faculty of Community and Health Sciences, University of the Western Cape, Cape Town, South Africa; 2 Department of Health and Rehabilitation Sciences, Faculty of Human Sciences, University of Cape Town, Cape Town, South Africa; 3 Department of Psychology, Faculty of Arts, Stellenbosch University, Cape Town, South Africa

**Keywords:** disability, healthcare access, equity, discrimination, South Africa, COVID-19, health systems research, human rights

## Abstract

People with disabilities, especially those living in low- and middle-income countries, experience significant challenges in accessing healthcare services and support. At times of disasters and emergencies, people with disabilities are further marginalised and excluded. During the coronavirus disease 2019 (COVID-19) pandemic, many people with disabilities are unable to access healthcare facilities, receive therapeutic interventions or rehabilitation, or gain access to medication. Of those who are able to access facilities, many experience challenges, and at times direct discrimination, accessing life-saving treatment such as intensive care unit admission and ventilator support. In addition, research has shown that people with disabilities are at higher risk of contracting the virus because of factors that include the need for interpersonal caregivers and living in residential facilities. We explore some of the challenges that people with disabilities residing in South Africa currently experience in relation to accessing healthcare facilities.

## Background

The protection of people with disabilities is enshrined within core legislative acts in South Africa, including *The Constitution* (1996) and *the Promotion of Equality and Prevention of Unfair Discrimination Act* (2000). Furthermore, South Africa was the second country to ratify the *United Nations Convention on the Rights of Persons with Disabilities* (UNCRPD) which speaks directly to the rights of people with disabilities to access information, transport, healthcare and the right to life.^1,2,3^ Despite these protections, there remain high levels of exclusion of people with disabilities in accessing healthcare. We sketch some of the key issues, with particular reference to the coronavirus disease 2019 (COVID-19) situation. These include challenges in accessing transportation, personal care, communication, therapeutic interventions, rehabilitation and medication; accessing intensive care unit (ICU) beds and ventilator support; and discriminatory triage policies.

## Disability and accessing healthcare

Across the globe, people with disabilities experience challenges in all aspects of life, including access to healthcare services, devices, required medication and support.^4,5,6^ These challenges are compounded in low- and middle-income countries (LMICs) where factors such as poverty, poverty-related diseases, inefficient healthcare systems, training and equipment, inaccessible transportation systems, corruption, political instability, and negative attitudes towards disability occur.^4,5,7,8,9^ These healthcare challenges aggravate the existing health conditions of most South Africans with disabilities.^10,11^ There exist multiple layers of inequality, as a result of the apartheid era, which further compound the challenges people with disabilities face on a daily basis.^12,13^ Despite the establishment of democracy in 1994, many black people with disabilities remain multiply disadvantaged, with black disabled females experiencing triple levels of discrimination based on their race, gender and disability.^14,15^ The difficulties facing people with disabilities living in rural areas are made worse through a lack of healthcare facilities and personnel, travel distances, inaccessible terrain and increased stigma attached to disability.^8,16^

In times of disaster and emergencies, many people with disabilities are further marginalised and excluded, experiencing an inability to access basic services, obtain information in an accessible format and receive therapeutic and/or medical interventions. With regards to COVID-19, additional factors such as pre-existing comorbidities, as well as communal living spaces such as residential or institutional facilities, further increase the risk of people with disabilities contracting the virus.^4^ Many have also experienced struggle and direct discrimination in accessing life-saving treatment such as ICU admission and ventilator support.^4,17^ These issues, which have placed people with disabilities at higher risk of contracting the COVID-19 virus during the current pandemic,^17,18^ are explored in depth in this article in both international and local contexts.

### Transport

A lack of accessible transportation is a significant barrier in accessing healthcare services for people with disabilities in South Africa.^3,16^ Railway networks are not accessible to wheelchair users, no assistance is provided to assist disabled passengers in accessing and using trains,^19^ and disabled passengers feel vulnerable because of safety concerns.^7,20^ In addition, there are no telecoil (TTY) facilities available for passengers using hearing aids, and many stations have poor signage and unclear loudspeaker systems.

The informal minibus taxi industry is the main provider of public transport in South Africa because of its availability and affordability.^21^ However, people with disabilities experience many challenges, including lack of assistance getting into and out of a taxi, being required to lift and carry their own wheelchairs on board, being charged an extra fare for their wheelchairs and concerns surrounding safety.^8,12,16^

A female wheelchair user said, in the context of COVID-19^22^:

It is so hard to convey to people that they won’t get Corona from touching my wheelchair. I show them I am sanitising my wheels, my chair. … when I use taxis I depend on people … to upload and offload me. But they don’t want to touch my chair … I see fear in people’s eyes. (p. 1)

During national lockdown Level 1 and Level 2, public transport was significantly reduced in order to assist in containing the spread of the virus. Public train services were suspended, a limited number of buses were permitted and minibus taxis were permitted to operate at limited capacity for limited hours. Transport was permitted for health workers and other workers employed in essential businesses and services, and people requiring essentials including food, medication and social grants.

Travel time limits were too restrictive for many people with disabilities who, as a result of their impairments and reliance on assistance from others, required a longer time to complete their morning hygiene routine, travel to purchase food and medical supplies and return home.

The limited public transportation severely affected the ability of caregivers to travel to assist people with disabilities. There was a lack of state communication, particularly from Department of Women, Children and Persons with Disabilities, the department responsible for supporting people with disabilities, regarding how, and from whom, permission could be obtained for caregivers to travel during lockdown.

### Personal assistance and caregivers

People with disabilities (and their families) carried a deep and constant fear that their caregivers may become sick and/or need to be quarantined.^23,24^ Unlike high-income countries where caregivers are paid for or procured via government structures and agencies, South Africans with disabilities are required to pay privately for caregivers or use their R1890 state disability grant for this purpose. There are very few structures and training providers such as carer agencies that provide qualified and trained substitute caregivers. As a result, the vast majority of caregivers have limited or no medical or healthcare training. Even if replacement caregivers were available, they would require training, and a careful handover, which would not be possible if immediate quarantine of regular caregivers was required.^24^

In the majority of cases, caregivers travel on public transport and interact with others at home, socially and whilst shopping when not on duty. Thus, COVID-19 testing is required before each shift change.^25^ The current cost of a single private COVID-19 test with 3-day return of result is R850, approximately half of the monthly disability grant and is beyond the reach of most South Africans with disabilities. Furthermore, private COVID-19 testing for caregivers is not covered by medical aid schemes of people with disabilities.

For some people with mental health conditions, loneliness and social isolation from caregivers has had a significant negative impact on their emotional and psychological well-being. It has been reported that the potential isolation from caregivers who assist with medication adherence, together with reduced accessibility to mental health services could lead to relapse.^26^

For one of the authors of this article, who has high-level quadriplegia, the reliance on carers and associated risks of contracting the virus from them is significantly high because of the nature of his disability and need for constant personal and intimate care. Not only is this extremely anxiety provoking, but because the author is unable to cough, contracting COVID-19 would most likely be fatal. His caregivers previously worked alternate shifts; however, in consultation with the caregivers, shifts have now been extended to one month to reduce the number of shift changes, travel time and costs involved. Not only is this physically and emotionally challenging, but COVID-19 has brought about additional and unbudgeted expenses. Furthermore, the author fears being admitted to hospital without his caregiver, as healthcare workers in hospitals may not have the skills, experience, training or time to provide him with the same necessary personal attention.

### Communication challenges

Current healthcare policy dictates that patients may not be accompanied by friends or family when accessing healthcare, which is a sensible prescription in general, but represents a significant obstacle for a number of people with disabilities. Many people who are deaf use sign language as their primary means of communication, and are unable to converse with healthcare workers without interpreters.^27,28^ For those who rely on lip reading, as the first author does, understanding healthcare workers wearing N95 and surgical masks is impossible. Furthermore, providing a patient history, or having to sign consent may be a significant obstacle, at times impossible for someone with severe intellectual or psychosocial disabilities, or on the autistic spectrum.

Information about COVID-19, how it is transmitted and prevention measures to be taken, has been released by the South African government across all media including television, radio and newspapers. However, not all of this information has been published in accessible formats, such as Braille, required by some people with disabilities.^4^ Additionally, only a few national broadcasts utilise sign language interpreters or subtitles to assist people with hearing impairments.

### Accessing therapeutic interventions, rehabilitation and medication

Studies in the United Kingdom, Italy and Iran reported critical scenarios for those with health conditions, such as strokes and spinal-cord injuries, as a result of the decrease of inpatient and specialised rehabilitation services, and restrictions on transport.^24,29,30^

Isolation and separation from families can have a negative impact on well-being, sometimes with devastating consequences. In China, a 17-year-old boy with cerebral palsy, Yan Cheng, died from starvation and lack of water as his father and brother were removed from their home and quarantined in a facility over 20 km away after being thought to have contracted COVID-19.^31,32^

In South Africa, most rehabilitation services have been temporarily cancelled, as far as we are aware. People with mental health conditions have had reduced access to mental health services because of the limited number of clinics providing services, with some having been periodically closed as a result of quarantine procedures.^26^ Special schools for disabled children, who require daily therapeutic services, have been closed since March 2020, leaving parents and caregivers with no or limited resources, and little or no training on how to assist their own children adequately in their homes.

### Accessing intensive care unit beds and ventilator support and discriminatory triage policies

During times of disaster and emergencies, when availability of resources is restricted, healthcare workers may be forced to make decisions as to who qualifies to receive life-saving healthcare. Emergency situations, such as the World Trade Centre attack (2001), and natural disasters, such as Hurricane Katrina (2005), have demonstrated the importance of having triage policies in place, and what challenges can occur if they are not established in emergency situations.^33,34,35,36^

Triage policies are fundamental in standardising the allocation of resources and care, as well as guiding healthcare workers in emergency practice.^4,37,38,39^ A range of triage tools are utilised across the globe and some differ within countries that have dual healthcare systems, such as South Africa, with government and private systems. Whilst triage policies are fundamental to effective emergency healthcare, it is important to ensure that they do not discriminate against any particular population group.^4^

Such discrimination is currently in evidence in London, UK (United Kingdom), where, during the COVID-19 pandemic, people with disabilities have reportedly been informed that they will not be admitted into hospital or receive life-saving emergency treatment if they become ill.^40^

In South Africa, triage policies relating to who can qualify for ICU admission and ventilator access currently discriminate against people with physical disabilities.^4^ Current healthcare triage policy aligns with the Frailty Assessment Score. Accordingly, a person with a Frailty Assessment Score of above 4 (anyone who is ‘mildly frail’ to ‘severely frail’) will be excluded from ICU access as well as ventilators if they become ill and resources are scarce. They will only be provided with oxygen therapy and urgent palliative care. This exclusion based purely on category of impairment raises questions relating to the value and worth placed on the lives of people with disabilities.

## Conclusion

People with disabilities, especially those living in LMICs, experience significant challenges in accessing healthcare services and support. During the current pandemic, many people with disabilities living in South Africa are unable to access healthcare facilities, including ICU beds and ventilator access, receive therapeutic interventions or rehabilitation, as well as medication.

The issues at play here are broad, and, indeed, structural. It is by now well established that the COVID-19 pandemic exposes and reproduces existing inequalities^41,42,43^ across the world. Debates on this issue emphasise issues of race and social class, and geographic and environmental issues.^44,45^ Despite the fact that there is a very well-established relationship between disability and poverty,^46^ the issue of disability in relation to COVID-19 is seldom mentioned as a consideration in debates about healthcare access and resource allocation.^47^ We have argued elsewhere that an aspect of the violence against people with disabilities lies in the continuing invisibility of disability in public discourse,^48^ leading to daily struggles for people with disabilities to gain access to employment, education and healthcare. Under pandemic conditions, a lack of considering disability as a public health equity issue may have profound consequences for the health, and the lives and livelihoods, of people with disabilities and those who care for them. At the heart of this issue is the question of whose lives are perceived to have worth – whose lives matter.
